# Analysis of Latin American Fertility in Terms of *Probable Social Classes*

**DOI:** 10.1007/s10680-020-09569-7

**Published:** 2020-11-03

**Authors:** Andrés Felipe Castro Torres

**Affiliations:** Max Planck Institute for Demographic Research, Konrad-Zuse-Straße 1 Office 259, 18057 Rostock, Germany

**Keywords:** Fertility, Social class, Latin America

## Abstract

Theories of demographic change have not paid enough attention to how factors associated with fertility decline play different roles across social classes that are defined multidimensionally. I use a multidimensional definition of social class along with information on the reproductive histories of women born between 1920 and 1965 in six Latin American countries to show the following: the enduring connection between social stratification and fertility differentials, the concomitance of diverse fertility decline trajectories by class, and the role of within- and between-class social distances in promoting/preventing ideational change towards the acceptance of lower fertility. These results enable me to revisit the scope of theories of fertility change and to provide an explanatory narrative centred on empirically constructed social classes (*probable social classes*) and the macro- and micro-level conditions that influenced their life courses. I use 21 census samples collected between 1970 and 2005 in Bolivia, Brazil, Chile, Colombia, Mexico, and Paraguay.

## Introduction

During the second half of the twentieth century, the total fertility rate for Latin America dropped from 5.9 to 2.6 children per woman (Guzmán 1996; Guzmán et al. 2006). This decline is associated with a decreasing demand for children, along with an increase in the ability of couples to practice birth control effectively. Five macro-developmental processes (forces of modernisation) are associated with these trends: (1) rising availability of modern contraceptive methods, (2) increasing educational attainment for women, (3) growing female labour force participation, (4) ongoing urbanisation (mainly due to rural-to-urban migration), and (5) improving mortality conditions (Bongaarts 2003; Castro Martin and Juarez 1995; Reher and Requena 2014). The mean age at first birth remained stable during this same period (Bongaarts et al. 2017; Palloni 1990). While less educated women were accelerating the transition to childbearing, highly educated women were postponing the first birth (Juarez and Gayet 2014). Consequently, across these five decades of rapid fertility decline, Latin American countries have exhibited wide fertility differentials and multiple fertility transitions by social strata. This variation is not well explained by the association between modernisation and lower/later fertility. A concentration on social structures and social classes is required to fully understand the specificities of the Latin American fertility transition.

Strong correlations between fertility (intensity and timing) and the forces of modernisation—at the individual and the country level—have led scholars to propose theories that tie lower and delayed fertility to improving material conditions (Hirschman 1994; Kohler 2010; Mason 1997; Myrskylä et al. 2009; Pesando and GFC team 2019). Other proposed theoretical frameworks have attributed these trends to non-material factors, such as institutional arrangements, social networks, systems of beliefs, cognitive structures, and social norms (Bachrach and Morgan. 2013; Cleland and Wilson 1987; McNicoll 1980). According to these theories, the extent to which people use material means to limit family size (e.g. delayed marriage, withdrawal, or modern contraception) can vary from one group to another depending on their cultural setting. Once a cultural change has occurred among one of the leading groups, it can spread to others through social interaction in a process referred to as ideational change (Bongaarts and Watkins 1996).

These theoretical frameworks have produced various explanations for long-lasting declines in family size, termed by van de Kaa as *anchored narratives* (1996). These *anchored narratives* continue to be widely used in examinations of contemporary and historical fertility change. They include the quality–quantity trade-off, the higher opportunity cost for women in the labour market, the reversal in the intergenerational flows of financial support, and the cultural transmission of low-fertility norms (Becker 1981; Bongaarts 2003; Caldwell 1982; Lee 2003; Shenk et al. 2013; Skirbekk 2008). It is my view, however, that none of these accounts is well suited to explain fertility variation and change in contexts of persistent levels of high economic and social inequality, such as Latin America. The main limitation of these explanations is that they do not pay enough attention to how modernisation forces affect the reproductive behaviour of individuals across social classes differently. Moreover, it is the operationalisation of social class belonging, typically proxied by one indicator (e.g. educational attainment, occupation), that limits the elaboration of more precise and nuanced narratives, i.e. narratives anchored in the reality of the social classes.

Demographers have long recognised the value of social class as an explanatory category. The importance of social class received considerable appreciation in studies of historical fertility change. As class structures evolved during the nineteenth and the first half of the twentieth centuries in capitalist economies, class differences in reproductive outcomes were documented using occupational categories as the preferred device for measuring class (Bourdieu and Darbel 1966; Coale and Watkins 1986; Schneider and Schneider 1996; Szreter 1996). The occupation of the male partner in a couple was considered a significant social marker of the couple’s social position, primarily because it reflected the man’s relationship to the means of production, following traditional Marxian class theory, but also, and perhaps more importantly, because it captured the confluence of the couple’s material conditions of existence, social status, and dispositions towards practices (e.g. modes of living, prestige, preferences, and family arrangements). In these studies, social classes served to explain variation in fertility because they captured the confluence between couples’ social position and their associated social dispositions.

Sociological approaches to conceptualise and measure social class have changed substantially since then (Wright 2015). The empirical observation of class-specific dispositions has gained substantial salience in contemporary studies of class inspired by the work of Pierre Bourdieu (Bourdieu 1996; Weininger 2005). Bourdieu argued that what defines a social class in a society is a set of historically situated relationships. What makes social classes an explanatory factor is the set of historical relations that define them. Consequently, there is no single social, economic, or demographic characteristic (variable) that can define a social class in a historically meaningful manner. This conceptualisation of class has two main strengths for the study of fertility in Latin America.

First, social class belonging becomes a multidimensional construct. This multidimensionality reflects the fact that high and persistent levels of economic and social inequality make social classes more dependent on the intersection of multiple socio-economic characteristics than on any single feature. Second, explanatory models of family and fertility variation provide an explicit connection between (multidimensional) social classes and social dispositions, i.e. between the material and the symbolic dimensions of social life (Johnson-Hanks et al. 2011 Introduction; Portes 2006). Because fertility is affected by factors that pertain to both realms, examining this connection enriches our explanations for between-class variations in fertility outcomes, and for how these patterns have changed over time.

This paper uses these two strengths of this conceptualisation of class to study changes in fertility across the cohorts of women born between 1920 and 1965 in six Latin American countries: Bolivia, Brazil, Chile, Colombia, Mexico, and Paraguay. These six countries had diverse trajectories of fertility decline and socio-economic development during the period of study (Fig. 5). The selected sample is also advantageous because it includes countries that are located at a wide range of latitudes, that have different population sizes (the three most populous countries of the region, but smaller countries as well), and that, due to data limitations, have been relatively understudied (Bolivia and Paraguay). Moreover, since the mid-1960s, these nations have differed in their approaches to implementing family planning programmes (FPPs) (Bongaarts and Sinding 2011, 2009; Parrado 2000). Chile, Colombia, and Mexico are known for having successful FPPs that have been strongly supported by national governments. In contrast, the governments of Paraguay, Brazil, and Bolivia have been less successful in providing reproductive and health services to women via FPPs, in part due to considerable resistance in the population (Carvalho and Brito, 2005; Martine 1996; Miller 2005). The regularity of patterns across classes among this variegated sample of countries is indicative of the potential generalizability of these results to societies with similar class structures.

## Class as a Multidimensional and Relational Construct

Individual-level studies of fertility decline in Latin America have focused on fertility differences stratified by socio-economic markers such as marital status, occupation, educational attainment, and place of residence (Adserà and Menendez 2011; Fussell and Palloni 2004; Itaboraí 2015; Martinez 1998; Palloni et al. 1996; Schkolnik and Chackiel 2004). However, the statistical categories of these markers cannot be seen as substitutes for social classes.

As modern societies become more complex and unequal, the relevance of configurations of socio-economic characteristics for social class differentiation increases, as well as the relevance of class differences for explaining social outcomes (e.g. fertility). The intersection of individuals’ socio-economic living conditions is better able than single categories to capture the context in which fertility decisions are made. The value of a social marker, such as occupational status, depends on the other socio-economic characteristics of the individual and his/her partner and the overall distribution of that marker across the population. In highly unequal societies, only specific configurations of social markers may exist as demographically significant groups. For example, despite the importance of secondary/college education for fertility postponement and decline, the contribution of secondary/college-educated women in rural areas to the overall fertility transition is minor: first, because they are a very small group (sometimes non-existent); second, because the mechanisms by which secondary/college education could reduce fertility vary between urban and rural areas: the relationship between educational attainment and fertility is context-dependent (National Research Council 1999).

To account for higher complexity and context dependency, social classes must be inductively defined, multidimensional, and relational. First, inductively defined classes mean that the grouping of individuals should be a result of data analysis, and not the application of a predefined partition (e.g. educational attainment groups). Second, when I refer to social class as multidimensional, I mean that class membership does not depend on a single characteristic, but rather on the intersection of characteristics. Third, when I refer to social class as relational, I mean that the value of a specific configuration of socio-economic markers depends on the prevalence of that configuration in society. Low-prevalence configurations can be observed among individuals in very privileged or very marginalised positions, which in turn determine the social relations that link these groups. For example, having a high school degree is less valuable, and not having a high school degree is more consequential, in urban than in rural areas, because in cities, the vast majority of the population have this level of educational attainment. Likewise, while not owning a dwelling is a clear marker of deprivation in rural areas because having access to land is essential to subsistence in the countryside, not owning a dwelling is generally considered less problematic in urban areas.

## Measurement and Interpretation of *Probable Social Classes*

Individuals’ social positions (social class) are determined by the intersection of their socio-economic attributes and how these socio-economic attributes distribute within the society, that is the primary theoretical premise of *probable social classes*. Once *probable social classes* have been identified, class differences in the level and the timing of fertility can be attributed to both their objective positions within the social space (between-class distances) and their associated dispositions (within-class distances). Applying this approach to individuals of successive birth cohorts allows *probable social classes* and fertility change to be attached to the historical variations encountered by each cohort (Smith 2019).

The explanatory power of this approach relies on the assumption that different material conditions produce (are linked to) *segmented rationalities* to use McNicoll’s (1980) terminology, class-specific *habitus* in Bourdieu’s (1996) terms, and situated mental *schemes* in Johnson-Hanks’ (2011) jargon. Recently developed socio-demographic theories have identified this link, along with its connections to differential behaviour and broader dynamics of power relationships and social inequality.

People similar to each other in social and economic positions will tend to be similar in the nature and type of materials available to them […]. This similarity comes both from the fact that they are likely to perceive and categorize materials in similar ways, as well as from common relations of power and inequality (Johnson-Hanks et al. 2011, p. 11).

Different positions imply different behaviour due to the combined action of material and non-material position-specific conditions. In addition, different locations in the social space reflect social inequality and power relationships, i.e. some positions are associated with positively valued social characteristics, while others are associated with negatively valued social features (privilege vs. non-privileged positions); and some positions exist (and persist) at the expense of other positions. The permanence over time of these positions can be called class consolidation, and the distribution of social agents among them can be called the social structure. A *probable social class* is a group of people closely located within the social structure.

Individuals’ positions can be derived empirically by applying geometric data analysis (GDA) techniques to individual data on occupation, position at work, housing conditions, educational attainment, access to basic services, and place of residence (material dimension). GDA can be used to identify the main configurations of socio-economic characteristics that distinguish social agents and to group them into *probable social classes* (Bourdieu 2005). To capture individuals’ material living conditions as well as aspects of their social status, the socio-economic characteristics that are included in the analysis should be as comprehensive as possible (Robson and Sanders 2009, Chapter 2).

The relative sizes of these classes across generations provide information on how social mobility and class consolidation occur within a given society. Small groups are made up of individuals in hard-to-reach positions, whereas large groups are made up of individuals in common living conditions. If the positions of large groups of socially disadvantaged people (lower classes) remain socio-economically distant from those of small groups of socially advantaged individuals (upper classes) across generations, social mobility is restricted. The stability of these social groups and the increasing distances among them over time reflect class consolidation.

This notion of social distance across social classes is very important for this study. Across Latin American societies, social distance implies low levels of interaction between societal groups due to the segregation by social class of residential spaces and educational systems. As socially close people tend to live in close physical proximity to each other, it may be assumed that they have higher levels of social interaction/exchange with each other than with socially distal people (Davis and Casis 1946; Portes 1971, 1989). Moreover, from the primary to the higher education level, there is a clear division between high- and low-class educational institutions (Balan 2013). Members of the upper classes are more likely to have access to competitive (often private) institutions, whereas members of the lower classes are more likely to attend low-quality institutions (Torche 2014).

## Data and Methods

### Sample Description

The analysis includes native-born women between the ages of 40 and 49 from 21 census samples of six Latin American countries with comparable socio-economic and social status variables: Bolivia, Brazil, Colombia, Chile, Mexico, and Paraguay.^1^ Only samples with information on the children ever born, the children who survived, and household identification variables are included (Minnesota Population Center 2015). These three types of variables are used to compute the outcomes of interest: mean children ever born by age 40 (complete fertility rate, CFR), mean age at first birth (MAFB), and mean age at last birth (MALB).

**Fig. 1:**
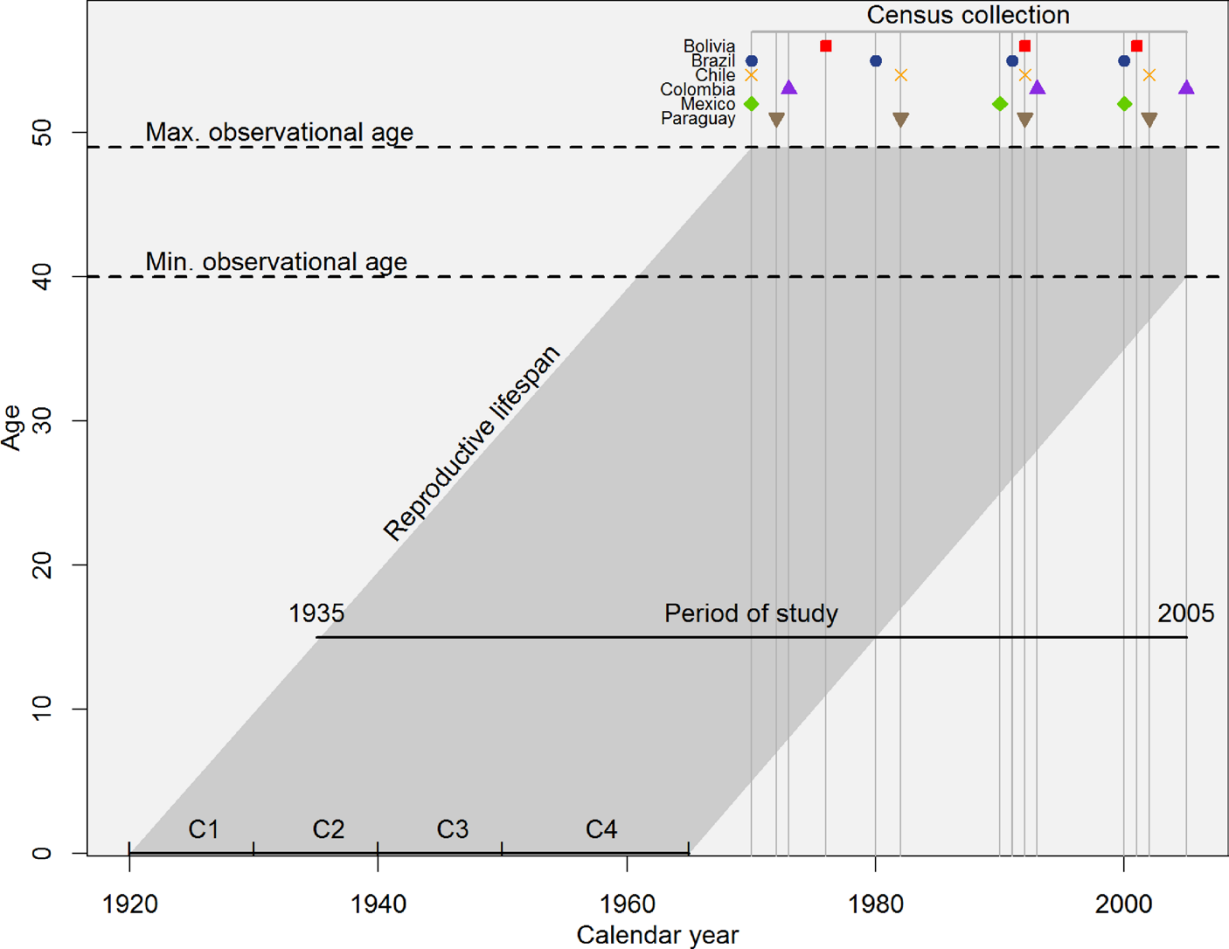
Reproductive lifespans of women in the analytical sample and exact year of data collection. *Note* The dark-shaded-grey parallelogram represents the lifespan of the birth cohorts included in this study. Vertical grey lines indicate the exact year of the census collection. Because the time between censuses varies within and between countries, the location of the cohorts (C1, C2, C3, and C4) is approximate

Figure 1 displays the exact years of the 21 censuses used in the analysis (grey vertical lines). As most of these data were collected during the first half of each decade from the 1970s to the 2000s, the women in the samples can be grouped into four ten-year birth cohorts: 1920–1929 (C1), 1930–1939 (C2), 1940–1949 (C3), and 1950–1965 (C4). People who were born in the same decade have a shared experience of historical time and are exposed to the same configuration of institutional arrangements (Ryder 1965, 1983). Assuming childbearing starts at age 15, the reproductive years of these women span the period 1935 to 2005.

Women in C1 and C2 entered reproductive ages when the socio-economic transformations associated with fertility decline were still in their early stages (1935–1950); indeed, some studies suggest Latin American and Caribbean countries experienced an increase in fertility during the post-war period (Reher and Requena 2014). In contrast, women in C3 and C4 entered reproductive ages when processes of mortality decline, urbanisation, educational expansion, access to modern contraception, and rising female labour force participation were accelerating (1950–1980), and consequently, fertility decline among these cohorts was more pronounced. Additionally, women in C4 can be seen as the population-level “daughters” of women in C1 and C2.

The couple is the unit of analysis primarily because, for the mid-twentieth century Latin America, social class is inextricably a property of the family. Also, it is at the couple level that fertility decisions are made. There are approximately 1.8 million couples in the analytical sample (Table 1).

**Table 1:** Sample size by country and birth cohort

Country	Birth cohort				Total
	C1	C2	C3	C4	
	1920–1929	1930–1939	1940–1949	1950–1965	
Bolivia	12,135		11,535	16,532	40,202
Brazil	1,45,084	1,70,883	2,62,189	3,38,384	9,16,540
Chile	23,869	28,863	41,641	60,224	1,54,597
Colombia	32,977		60,119	53,259	1,46,355
Mexico	10,760		1,92,566	2,68,736	4,72,062
Paraguay	6004	7149	10,991	14,600	38,744
Total	2,30,829	2,06,895	5,79,041	7,51,735	17,68,500

*Note* The sample includes native-born women ages 40–49 living with a partner or spouse at the time of the census and with complete information on all socio-economic and fertility-related variables

Having the couple as the unit of analysis requires restricting the sample to women who were living with a spouse or a partner at the time of the census. Thus, the samples used are not representative of all women across the six countries. Specifically, the samples only account for the subset of the women in these populations who survived to ages 40–49, were married or in a union, and whose partner/spouse was present at the time of the census. These women represented 62% of ever-married women and contributed 63% of total births recorded by the censuses.^2^

### Calculation of the Mean Age at First and Last Birth

I calculate women’s age at first and last birth by subtracting, respectively, the ages of the oldest and youngest child from the age of the mother. The children need to be part of the household in order to be linked to their mothers. If at least one child is absent, the ages at first and last birth of a woman are uncertain.

To partially correct this bias, I impute the age at first birth of women with at least one child not present in the household using the age at first birth for women whose children are all enumerated by the census. Because the imputation uses information from all observed births, I refer to these cases as incomplete (at least one child is absent) and complete birth histories (all children are present). The main assumption of this imputation is that similarity in the timing of the observed births implies a similar age at first birth (Miranda-Ribeiro et al. 2009). I do not implement any correction for the age at last birth, as it is less sensitive to these issues. The last birth is closer in time to the date of data collection, and the youngest child is more likely than the oldest child to be part of the household at the time of the census. I have computed estimates of the mean age at first and last birth using data from the Demographic and Health Surveys for some of the countries and have concluded that the results are consistent.

Consider a woman who had four children but only three of whom are linked to her in the census. This woman has an incomplete birth history; I know her age at birth for three of her four children, but I cannot be certain about her age at first birth. Consequently, I impute her age at first birth using the age at first birth of a woman of the same age, with four children, whose age at first birth is certain (complete birth history), and whose timing of births is very similar to the timing of the three observed births of the woman with the incomplete birth history. This similarity can be measured in relative terms as a matching score according to the number of births that occurred at the exact same ages for two given women. For the example of two women with four children, the matching score will be 100% if these women had three births at exact same ages, 75% if this is the case for only two births, 25% if this is the case for only one birth, and 0% if their ages at birth do not coincide at all. The higher the similarity, the more realistic the imputation.

To assess the effectiveness of this procedure, Table 2 presents the number of complete and incomplete birth histories by the women’s parity levels. The matching score organises incomplete birth histories into five groups ranging from birth histories that do not match at all to birth histories that match almost perfectly (75–100%). Percentages by row are below the absolute numbers.

**Table 2:** Birth histories distribution by parity level, completeness, and matching score

Parity level	Birth histories						Total
	Complete	Incomplete					
		Relative matching score (%)					
		Zero	(0–25]	(25–50]	(50–75]	(75–100]	
Zero	57,672	–	–	–	–	–	57,672
	100	–	–	–	–	–	100
One	91,370	19,588	–	–	–	–	1,10,958
	82	18	–	–	–	–	100
Two	2,17,649	23,287	–	–	–	55,576	2,96,512
	73	8	–	–	–	19	100
Three	1,83,711	19,100	–	2182	–	1,22,921	3,27,914
	56	6	–	1	–	37	100
Four	89,068	12,223	–	1663	9225	1,21,148	2,33,327
	38	5	–	1	4	52	100
Five to six	73,150	13,482	109	4600	36,169	1,77,218	3,04,728
	24	4	0	2	12	58	100
Seven and more	40,938	28,279	1748	17,400	95,804	2,53,220	4,37,389
	9	6	0	4	22	58	100
Total	7,53,558	1,15,959	1857	25,845	1,41,198	7,30,083	17,68,500
(%)	42.6	6.6	0.1	1.5	8.0	41.3	100

*Note* A birth history is assumed to be complete when the number of children ever born reported by a woman equals the number of own children present in the household at the time of the census. Information on children ever born comes from the variable: CHBORN. Own children are identified using the variables: MOMLOC and STEPMOM. Birth misreporting is ignored because it is unlikely to be consequential for the results

Overall, 43% of the birth histories are complete, 41% match with a score above 75%, and less than 7% of the birth histories do not match at all. Birth histories that are complete or are almost perfectly matched together account for all but 16% of the total sample—cases that could conceivably induce an upward bias in the mean age at first birth. This is not problematic for the results for two interrelated reasons. First, this percentage is low and concentrated in high parities, meaning that the mean age at first birth is more likely to be overestimated among high-fertility women. Second, this upward bias will be more substantial among groups characterised by high infant mortality, early childbearing, early departure from the parental household, and the tendency to misreport the total number of births. These four conditions are more common among socio-economically disadvantaged couples than among the socially privileged (Fussell and Palloni 2004). In consequence, differences in the mean age at first birth by social class are likely to be lower-bound estimates of the actual class differences.^3^

### Explanatory Variables, Geometric Data Analysis, and Clustering

I use GDA and clustering techniques to construct country- and cohort-specific social spaces and *probable social classes*. GDA makes it possible to summarise the multivariate correlations among a set of variables (called active variables) into factorial coordinates, i.e. into a reduced number of numerical variables. Because all active variables in this analysis are categorical, the analysis is termed multiple correspondence analysis (MCA) (Greenacre and Blasius 2006; Le Roux and Rouanet 2004).

Active variables measure the socio-economic conditions and social status of couples as comprehensively as possible while keeping cross-national comparability. The maximum number of variables that can be harmonized for all the census samples is nine. These nine active variables are educational attainment (the woman’s and the partner’s), place of residence, ownership of the dwelling, position at work (the woman’s and the partner’s), economic sector (the partner’s), television and electricity, and type of water supply. The variables are re-coded to avoid categories with relative frequencies below 2% and to have a similar number of categories across variables (refer to Table 2 for the complete list of variables and categories). These two features help to ensure that the factorial coordinates are not biased (Lebart et al. 1997). Couples with similar values along the factorial coordinates are considered socially close. Couples with divergent coordinates are considered socially distal.

Factorial coordinates have technical characteristics that make them suitable for use in identifying *probable social classes*. Factorial coordinates maximise the proportion of explained variance hierarchically. The first factorial coordinate summarises the highest proportion of the variance among active variables like a regression line maximises the R^2^. (Conveniently, however, MCA techniques do not require the definition of an outcome variable). This proportion decreases monotonically among the remaining factorial coordinates. When the correlation among variables is strong, a relatively small number of factorial dimensions comprise a large proportion of the total variance.

Factorial coordinates have two theoretical characteristics. First, they determine the objective (material) position of couples in a given social space while accounting for both micro- and macro-level characteristics, i.e. the couple-level socio-economic features and the overall distribution of those features in the population. Second, these data-driven/multidimensional positions are associated with class-specific dispositions. This assumption holds for this paper because the women in the sample are 40 years and older, and the nine active variables measure the current living conditions and the life trajectories of the couples. For example, lacking educational credentials at age 40 signals a life trajectory of restricted access to cultural resources; likewise, lacking an owned dwelling at that age signals limited asset accumulation. While individuals can experience social mobility or improve their socio-economic conditions after age 40, it may be assumed that after this age, these two aspects of life, like fertility, become increasingly fixed and generally reflect the overall trajectory of an individual’s life.

For the cluster analysis, between-couple Euclidean distances are computed using the first four factorial dimensions (i.e. the first four coordinates yielded by the MCA, see the justification in the next section). These social distances are organised in a pair-wise matrix (*D*). The generic term of this matrix *d*_*ij*_ measures the social dissimilarity between couples *i* and *j*. I apply hierarchical and non-hierarchical clustering techniques to this matrix to identify *probable social classes*. I start with a hierarchical method: the Ward method (Kaufman and Rousseeuw 2005, Chapter 4). The main advantage of this approach is that the number of clusters can be determined based on the proportion of explained variance of different cluster solutions (Studer 2013). The higher the proportion of explained variance, the better the cluster solution. This proportion is noted *R*^*2*^*(c)*, where *c* is the number of clusters. Because *R*^*2*^*(c *+ *1) *>* R*^*2*^*(c)* for all possible values of *c*, an adequate cluster solution can be identified by combining two criteria: the percentage of explained variance by a given partition and the marginal increase in the explained variance between two subsequent partitions. A high value of *R*^*2*^*(c)* and a small marginal increase between two subsequent cluster solutions (*c* and *c *+ *1*) are indicative of an adequate solution. After selecting a number of clusters using these two criteria, I implement a non-hierarchical clustering method: The *K*-means algorithm. This algorithm consolidates the hierarchical grouping assuring more consistent clusters (Pardo and Del Campo 2007).

## Results

**Fig. 2:**
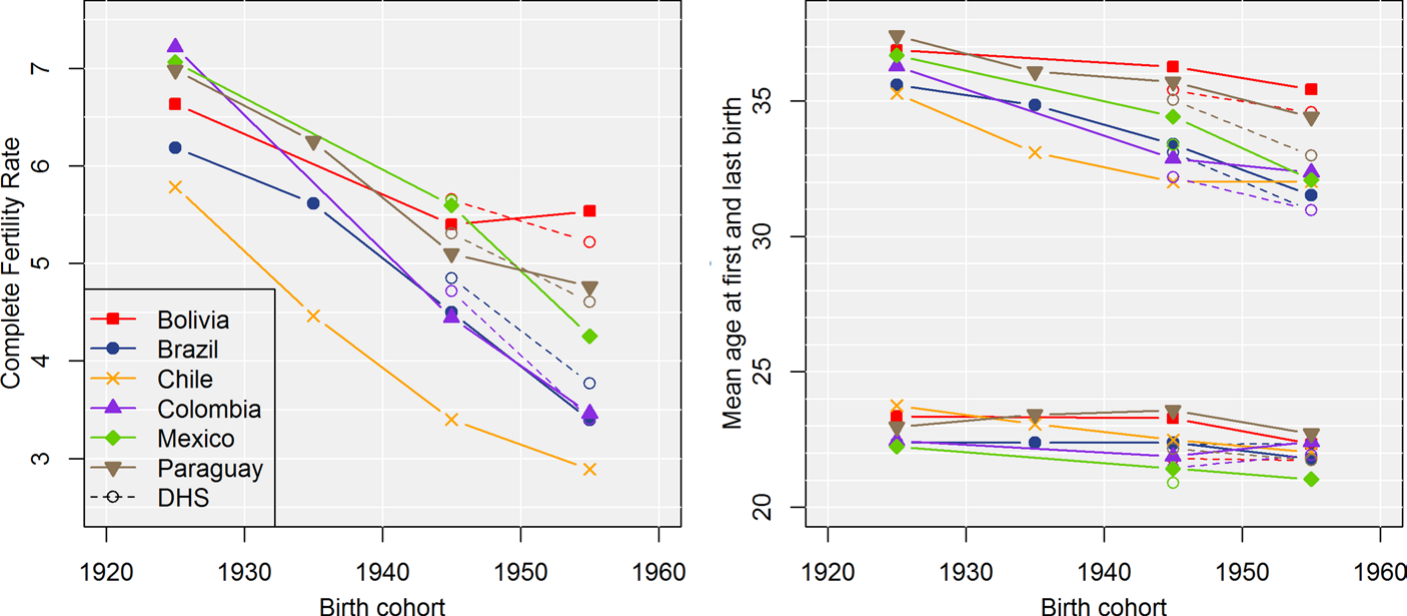
Fertility indicators by country and birth cohort. *Note* Census data from IPUMS-I (solid lines) and Demographic and Health Surveys (DHS, dotted lines). DHS figures are obtained using full retrospective information on childbearing

Fertility decline across cohorts was widespread, but unequal, across countries. As Fig. 2 shows, the decrease in the complete fertility rate between the first and last cohort varied between 1.1 children in Bolivia and 2.9 children in Chile. By contrast, the mean age at first birth (MAFB) displayed only small changes. The mean age at last birth (MALB) declined substantially—by at least three years—between the first and the last cohort, except in Bolivia.

High levels of income, land tenure, and wealth inequality are characteristic of the robust stratification systems in Latin American (Morley 2001; Portes 1985; Portes and Hoffman 2003; Williamson 2010). These social and economic inequalities have two key features. First, there is a pronounced and self-perpetuating concentration of resources among the upper classes, most of whom live in capital cities. Second, the role of educational attainment in reducing inequality and facilitating social mobility is small (Hoffman and Centeno 2003; Torche 2014). Consequently, most of the changes in the class structure of Latin American societies during this period resulted in the enlargement of the lower classes. It has, for example, been observed that for the rural poor and for rural-to-urban migrants, the expected societal benefits of urbanisation, access to contraception, higher educational attainment, and female labour force participation have produced little, if any, actual return (García and de Oliveira 2011). The socio-economic profiles of the studied cohorts reflect these societal changes.

Table 3 shows the socio-economic profiles of couples of the first and the last cohorts studied. In all six countries, the proportions of men and women with no education declined over time to levels below 60%. The shares of the population with primary education grew for both sexes, but having a university degree remained uncommon. Despite educational improvements, more than 50% of the individuals of the last cohort had no more than a primary-level education.

**Table 3:** Socio-economic profiles for two cohorts in six Latin American countries—all values are percentages and add to 100 per variable

Country	Bolivia		Brazil		Chile		Colombia		Mexico		Paraguay	
Birth cohort	C1	C4	C1	C4	C1	C4	C1	C4	C1	C4	C1	C4
*Woman’s educational attainment*												
No education	87	51	92	56	53	15	70	35	80	37	83	42
Primary	10	25	4	18	38	46	27	39	18	44	12	40
Secondary	3	19	4	18	8	35	3	17	2	12	4	14
University	–	4	–	8	–	5	–	9	–	7	–	5
*Man’s educational attainment*												
No education	79	34	90	58	48	14	67	39	77	33	80	41
Primary	16	36	5	18	40	43	27	36	19	42	14	41
Secondary	6	22	5	15	12	36	3	14	4	11	6	12
University	–	9	–	9	–	8	2	10	–	14	–	6
*Couple’s place of residence*												
Capital city	13	20	28	33	48	55	14	12	16	11	18	10
Rural areas	66	36	43	18	25	11	39	33	40	19	62	40
Urban areas	21	45	29	49	28	34	47	55	44	70	20	50
*Ownership of the dwelling*												
Owned	83	76	67	81	59	77	67	65	70	86	88	88
Renting	9	15	16	10	21	14	21	23	30	14	5	5
Provided	8	9	6	9	20	10	9	9	–	–	–	–
Occupied	–	–	12	–	–	–	3	4	–	–	6	6
*Electricity|Television*												
Yes|Yes	–	63	29	90	90	96	–	77	36	91	9	80
Yes|No	–	8	20	5	5	2	–	17	27	6	10	13
No|No	–	29	50	5	5	2	–	6	37	3	81	7
Yes|n.a.	30	–	–	–	–	–	61	–	–	–	–	–
No|n.a.	70	–	–	–	–	–	39	–	–	–	–	–
*Water supply*												
Piped exclusive	13	40	37	85	64	94	58	81	44	67	7	45
Piped shared	21	35	5	5	16	4	10	–	22	24	5	26
No piped water	67	26	58	9	20	2	32	19	34	8	88	29
*Woman’s position at work*												
Self-account	8	34	3	14	4	7	3	6	3	11	8	20
Employee(r)	4	14	6	30	8	29	5	25	4	19	4	18
Inactive	88	51	91	56	88	64	93	70	92	70	87	62
*Man’s position at work*												
Employer	2	5	4	6	4	6	16	–	8	5	4	6
Self-account	70	57	50	40	25	22	28	24	35	33	69	60
Employee(r)	28	38	46	55	70	72	56	76	57	62	27	35
*Man’s economic sector*												
Agriculture	65	35	49	22	30	16	44	36	49	20	61	34
Manufacturing	7	11	11	13	18	15	14	7	16	17	11	11
Construction	6	11	8	12	10	12	8	9	6	12	6	12
Services	12	22	14	30	18	30	15	32	14	28	10	18
Sales	4	12	12	14	15	17	12	9	10	14	8	18
Clerical and education	5	9	7	9	9	10	7	6	5	9	4	7

*Not*e *C1* 1920–1929, *C4* 1950–1965 as in Fig. 1. Variables’ categories correspond exactly to those used in the multiple correspondence analyses

The effects of urbanisation and improvements in living conditions clearly differentiate these two cohorts. More than half of the couples in the last cohort were living in an urban area, owned a dwelling, and had access to electricity, a television, and piped water (exclusive or shared). However, the potential societal benefits associated with urbanisation did not materialise for the entire population, in part because cities did not have the level of industrialisation that would enable rural migrants to find jobs that offer upward mobility. Moreover, these migrant populations lacked the training to enter highly skilled occupations or to develop new businesses (Portes 1989). Studies on Latin American economic development have suggested that the decrease in agricultural production associated with migration to urban areas was not beneficial, as these countries started to import food that could have been produced internally (Escobar 2007; García-Nossa 1980, 1981). The distribution of the labour force by sectors across cohorts reveals the extent of these changes (last rows in Table 3).

Across these cohorts, the share of men occupied in agriculture decreased, whereas the share of men employed in the service industry went up. These changes reflect the general shift in national economies towards the tertiary sector as plans for industrialisation largely failed, and efforts to implement the import-substitutions model were unsuccessful (Baer 1972; Bethell 1998). While the share of workers in manufacturing grew in Bolivia and Brazil, it declined in the other four countries. The construction sector grew in all six countries, along with the sales and the public administration and education sectors. The last category (Clerical and education) employed the smallest share of the labour force overtime in all of the studied countries. Female labour force participation rose in all six countries, but more than half of the women in the last cohort reported being out of the labour market at the time of the census. Given the disproportionate representation of women in the informal economy and in non-remunerated occupations such as care work, these latter indicators should be taken with care. Especially for the lower classes, this high prevalence of formal labour force inactivity does not mean women did not participate in production activities.

**Fig. 3:**
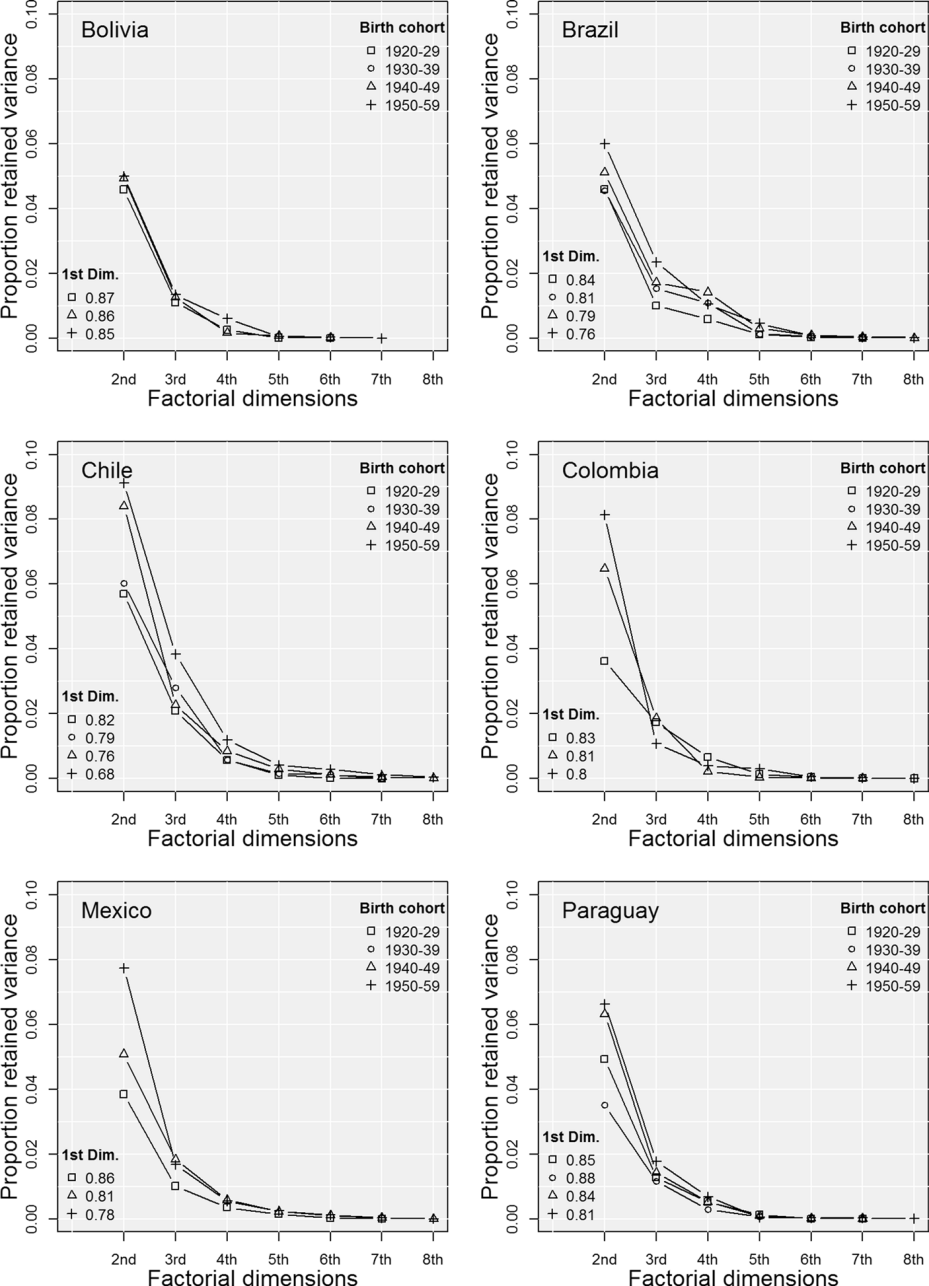
Proportion of explained variance across factorial dimensions. *Note*: The number of factorial dimensions equals the number of categories (Table III) minus the number of variables (9). Proportions are calculated according to Greenacre and Blasius’ (2006) formula

Across these cohorts, positively and negatively valued individual-level social markers were strongly correlated. At the aggregate level, these correlations consolidated sharply unequal social classes. It was only after the 1990s that increasing inequality trends underwent some reversals. The high proportion of variance accounted for by the first factorial coordinates of the MCAs reflects these enduring correlations. Figure 3 displays these proportions for the first eight factorial dimensions in each of the 21 MCAs. There is one panel per country, and the proportion for the first dimension is written as a label to facilitate visualisation.

Figure 3 shows that for all birth cohorts, the first four factorial dimensions accounted for more than 83% of the total variance of the active variables (min: 83% for C4 in Chile, max: 93% for C1 in Bolivia). Given these high proportions of explained variance, these four factorial dimensions are a good summary of couples’ socio-economic and social status conditions, and they can be used to perform a cluster analysis. In addition, Fig. 3 also shows that the first two factorial dimensions were very dominant. While the first accounted for more than 72% of the variance, the second one accounted for at least 55% of the remaining variability.^4^

This concentration of explained variance suggests that Latin American social spaces are rigidly structured, as the couples’ socio-economic and social status characteristics were strongly correlated. The variables that contributed the most to the first factorial dimension were those associated with the availability of economic resources: namely place of residence, access to basic services (electricity, television, and water supply), and occupation. The variables with the largest contributions to the second dimension were the educational attainment of women and their partners (cultural resources). These differential contributions mean that there were two main “independent” factors that were shaping the social structure within each country: namely economic and cultural resources. However, the distinction between these two types of resources requires a caveat. In Latin American societies, there is no social class with low economic capital and high cultural capital. While keeping this in mind and for the sake of conciseness, I will refer to the first dimension as an indicator of economic capital and the second dimension as an indicator of cultural capital.

The proportion of retained variance in the first dimension declined across cohorts, whereas that in the second dimension increased. This means that over time, the role of cultural resources in differentiating the social classes relative to economic conditions increased. The proportion of explained variance in the first dimension was above 0.8 in all six countries for the first cohort, but varied between 0.68 in Chile and 0.85 in Bolivia for the last cohort. These changes reflect two aspects of societal transformation: first, an increase in the relative importance of cultural resources in structuring social spaces; and, second, a higher level of heterogeneity across cohorts in couples’ socio-economic conditions, and especially in their educational attainment levels. Full representations of the MCA outputs, including the distributions of variables and couples along the first two factorial dimensions, are available in Figs. 8 to 13.

The cluster analysis, based on the four-coordinate social distance matrices (83–93% of the total variance), provides further confirmation of the dual dynamics of improving socio-economic conditions and the endurance of social stratification. Three main results applied to all six countries: first, the number of social classes was very stable; second, the relative sizes of the classes and their variations over time (class consolidation) were similar; and third, the social distances between classes were substantial and persisted across cohorts.

In all cases, five clusters accounted for at least 55% of the total variance of the social distances among couples. The marginal increase in this percentage between clusters 5 and 6 was less than seven percentage points (refer to Fig. 7). The consistency of these results across the studied countries and cohorts suggests that these Latin American societies remained stratified over time across a relatively stable number of groups. I have labelled these classes using terms that refer to positions in space: lowest, low, lower middle, upper middle, and upper. Given the overlapping nature of these classes, I have chosen to use the expression “low classes” (with a lower-case “l”) to refer to the lowest, low, and lower-middle classes; the expression “middle classes” (lower-case “m”) to refer to the lower-middle and upper-middle classes, and the expression “upper classes” (lower-case “u”) to refer to the upper-middle and upper classes.

As Table 4 shows, the countries’ class composition varied widely over time among the lower classes, which signals that class consolidation, i.e. the changes in the sorting of couples into different class sections over time, mainly occurred within the lower layers of the stratification system. The relatively smaller changes among the upper-class groups signal the reverse. Between the first and the last cohort, the proportion of couples in the lowest class diminished in all six countries, and especially in the three countries with relatively high levels of development (46–11% in Brazil, 24–11% in Chile, and 37–7% in Mexico). In the last cohort, the share of couples in the low class was above 20% in all six countries except Colombia (13%). The share of couples in the lower-middle class increased across cohorts and accounted for at least 20% of couples in the last cohort (min: 19.6% in Chile, max: 32.5% in Mexico). In all six countries, the share of couples in the low class comprised at least 62% of the total.

**Table 4:** Marginal distribution of *probable social class* for the first and the last cohort

Probable social class	Country											
	Bolivia		Brazil		Chile		Colombia		Mexico		Paraguay	
Birth cohort	C1	C4	C1	C4	C1	C4	C1	C4	C1	C4	C1	C4
Lowest	61.0	32.7	45.7	10.7	24.1	10.9	39.5	29.8	37.0	6.8	61.8	17.9
Low	12.5	22.3	20.6	30.4	23.4	37.0	13.2	13.4	30.2	22.9	19.9	25.1
Lower middle	11.3	23.4	17.4	28.2	17.6	19.6	22.0	29.5	12.7	32.5	8.9	34.7
Upper middle	10.9	13.7	10.5	19.4	26.0	23.1	19.5	16.9	15.4	22.2	5.0	15.7
Upper	4.3	8.0	5.7	11.4	8.8	9.3	5.7	10.3	4.7	15.7	4.4	6.6
Total	100	100	100	100	100	100	100	100	100	100	100	100

*Note* All values are column percentages. *C1* 1920–1929, *C4* 1950–1965 as in Fig. 1

The shares of couples in the upper-middle classes were smaller and less variable in terms of size. Across cohorts, they tended to either increase or remain stable at relatively low levels (min: 13.7% in Bolivia, max: 23.1% in Chile). The proportions of couples in the upper class varied less across countries in the first cohort and increased over time. In all six countries, the upper class was small, comprising less than 6% of the couples in the first cohort and 16% of the couples in the last cohort.

**Fig. 4:**
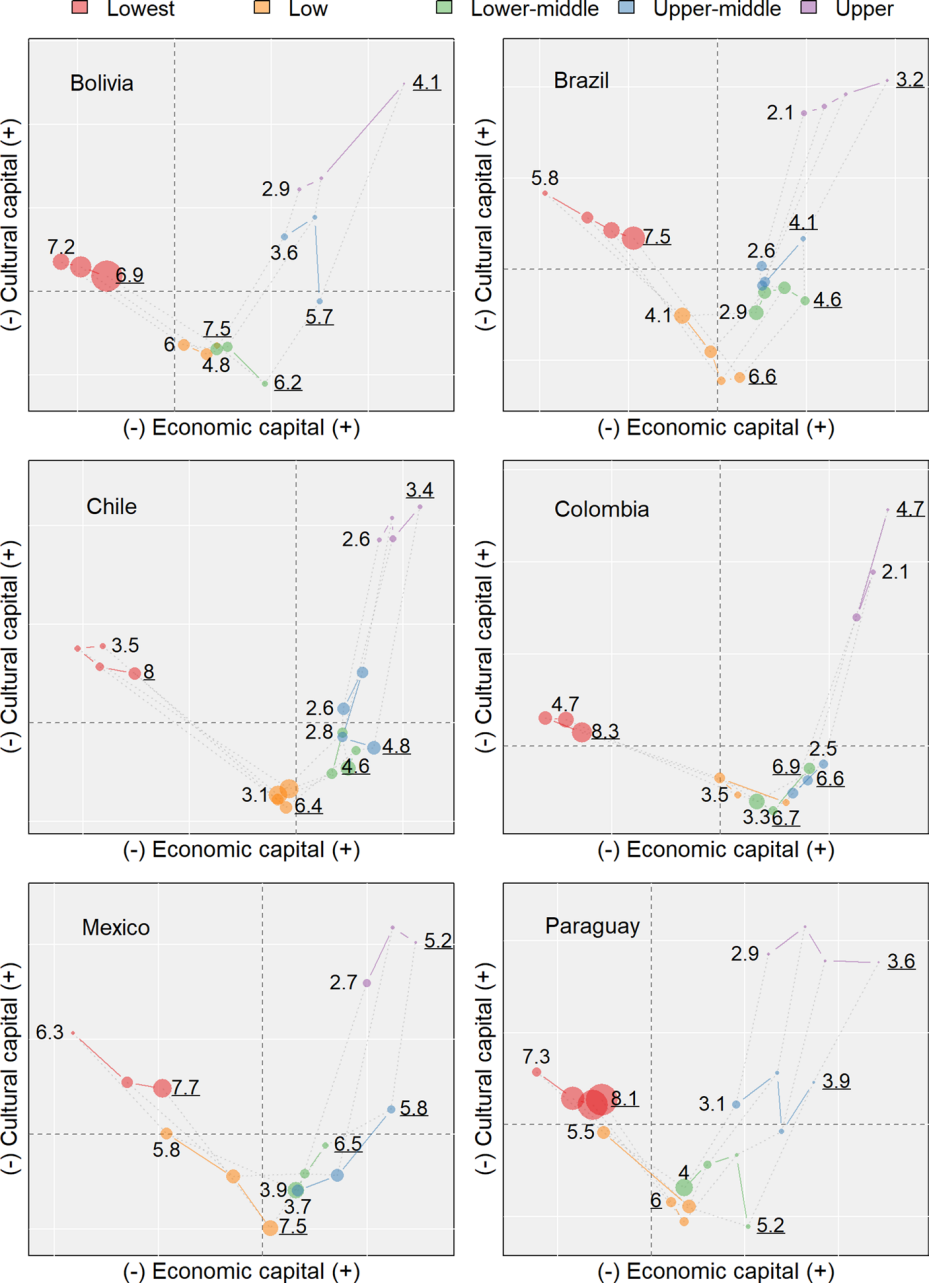
Social spaces, *probable social classes*, and complete fertility across cohorts. *Note:* The point’s size is proportional to the number of couples. Dotted lines represent between-class social distances. Background lines are separated by one standard deviation. Numbers correspond to the complete fertility rate of the first (underlined) and last cohort (C1 and C4))

**Fig. 5:**
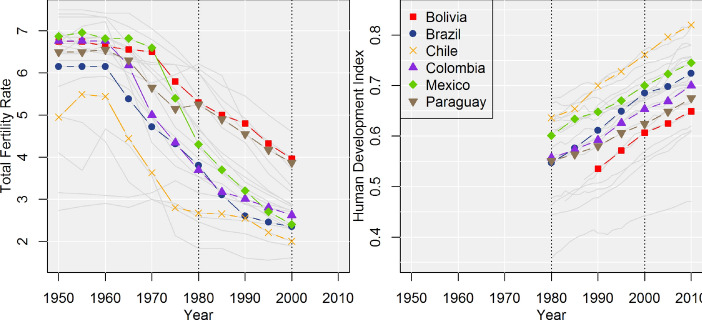
Total fertility rate and Human Development Index (HDI) for selected Latin American countries. *Note* Labelled countries are included in this paper. Fertility and HDI data come from the United Nations Economic Commission for Latin America and the Caribbean, and the United Nations Development Program, respectively. Countries in grey: Argentina, Belize, Costa Rica, Cuba, Dominican Republic, Ecuador, El Salvador, Guatemala, Haiti, Honduras, Nicaragua, Panama, Peru, Puerto Rico, Uruguay, and Venezuela

**Fig. 7:**
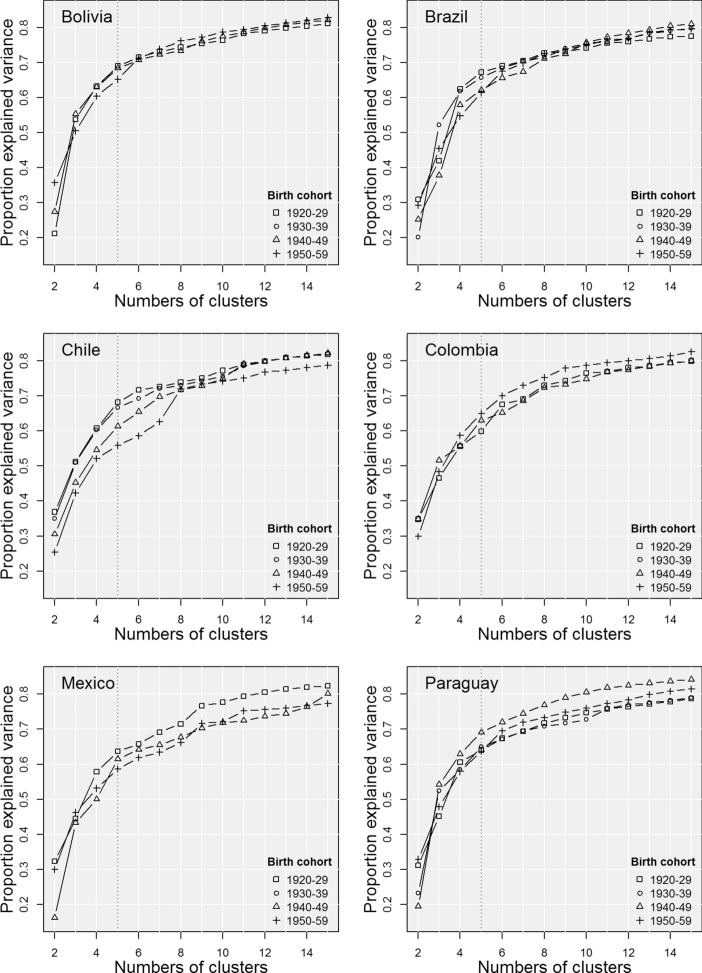
Proportion of explained variance for several numbers of clusters. *Note* The proportion of explained variance is computed as the ratio of the sum of squared distances within clusters and the total sum of squared distances according to Studer (2013)

**Fig. 8:**
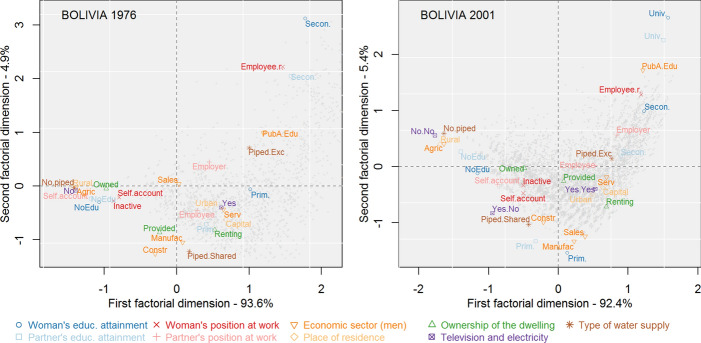
Variables’ correlation and couples’ distribution along the first two factorial coordinates—Bolivia. Note: Proximity between categories implies positive correlation; distance between categories implies the reverse. Grey *markers represent couples. Educational attainment: No education (NoEdu), Primary education (Prim), Secondary education (Secon.), University education (Univ.), Water source: Non-piped (No.piped), Piped shared (Piped.Shared), Piped exclusive (Piped.Exc), Position at work: Employee or employer (Employee.r), Economic sector (men): Agriculture and mining (Agric), Construction (Constr), Manufacturing (Manufac), Sales (Sales), Services (Serv), Public administration and education (PubA.Edu)

**Fig. 9:**
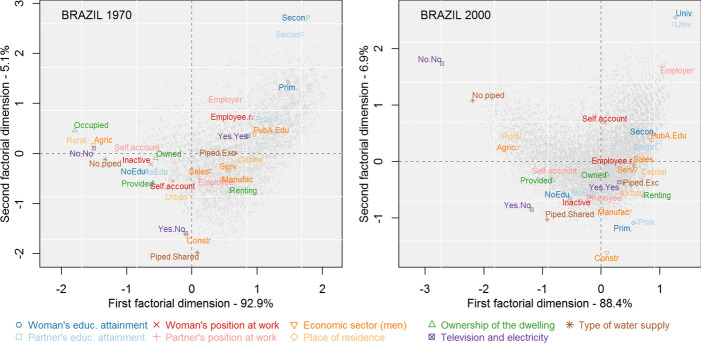
Variables’ correlation and couples’ distribution along the first two factorial coordinates—Brazil. Note: Proximity between categories implies positive correlation; distance between categories implies the reverse. Grey *markers represent couples. Educational attainment: No education (NoEdu), Primary education (Prim), Secondary education (Secon.), University education (Univ.), Water source: Non-piped (No.piped), Piped shared (Piped.Shared), Piped exclusive (Piped.Exc), Position at work: Employee or employer (Employee.r), Economic sector (men): Agriculture and mining (Agric), Construction (Constr), Manufacturing (Manufac), Sales (Sales), Services (Serv), Public administration and education (PubA.Edu)

**Fig. 10:**
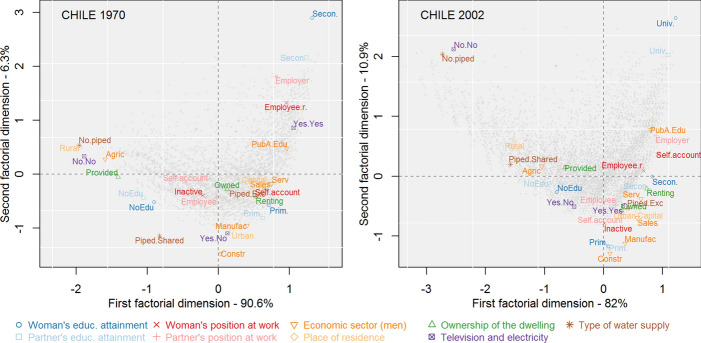
Variables’ correlation and couples’ distribution along the first two factorial coordinates—Chile. Note: Proximity between categories implies positive correlation; distance between categories implies the reverse. Grey * markers represent couples

**Fig. 11:**
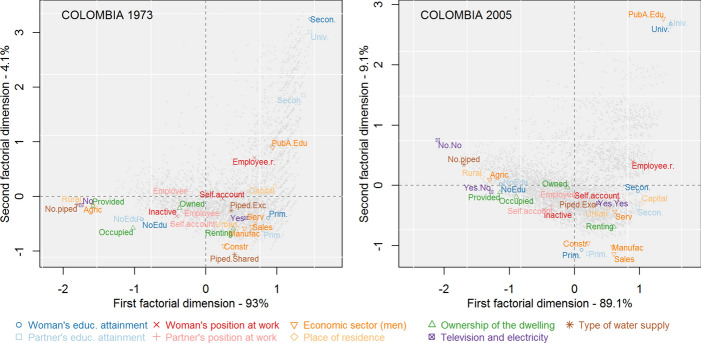
Variables’ correlation and couples’ distribution along the first two factorial coordinates—Colombia. Note: Proximity between categories implies positive correlation; distance between categories implies the reverse. Grey * markers represent couples. Educational attainment: No education (NoEdu), Primary education (Prim), Secondary education (Secon.), University education (Univ.), Water source: Non-piped (No.piped), Piped shared (Piped.Shared), Piped exclusive (Piped.Exc), Position at work: Employee or employer (Employee.r), Economic sector (men): Agriculture and mining (Agric), Construction (Constr), Manufacturing (Manufac), Sales (Sales), Services (Serv), Public administration and education (PubA.Edu)

**Fig. 12:**
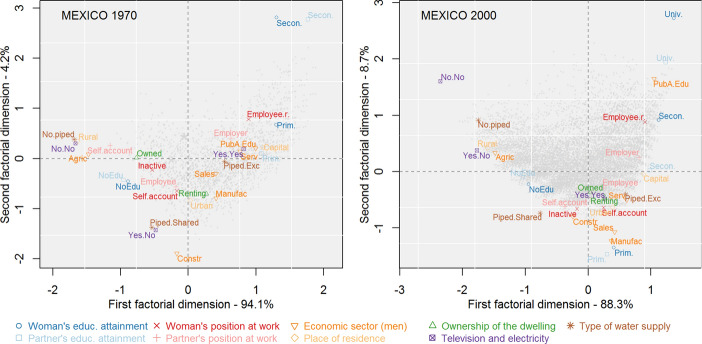
Variables’ correlation and couples’ distribution along the first two factorial coordinates—Mexico. Note: Proximity between categories implies positive correlation; distance between categories implies the reverse. Grey * markers represent couples. Educational attainment: No education (NoEdu), Primary education (Prim), Secondary education (Secon.), University education (Univ.), Water source: Non-piped (No.piped), Piped shared (Piped.Shared), Piped exclusive (Piped.Exc), Position at work: Employee or employer (Employee.r), Economic sector (men): Agriculture and mining (Agric), Construction (Constr), Manufacturing (Manufac), Sales (Sales), Services (Serv), Public administration and education (PubA.Edu)

**Fig. 13:**
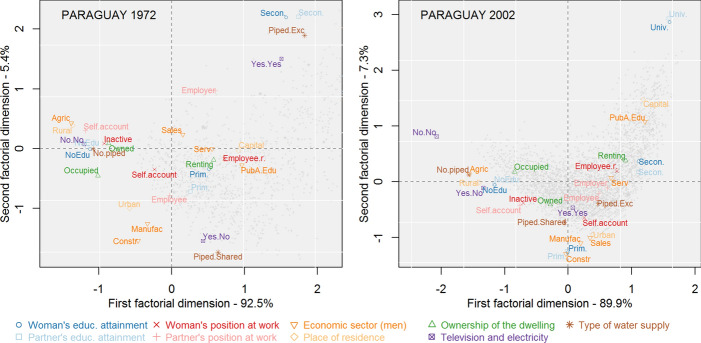
Variables’ correlation and couples’ distribution along the first two factorial coordinates—Paraguay. Note: Proximity between categories implies positive correlation; distance between categories implies the reverse. Grey * markers represent couples. Educational attainment: No education (NoEdu), Primary education (Prim), Secondary education (Secon.), University education (Univ.), Water source: Non-piped (No.piped), Piped shared (Piped.Shared), Piped exclusive (Piped.Exc), Position at work: Employee or employer (Employee.r), Economic sector (men): Agriculture and mining (Agric), Construction (Constr), Manufacturing (Manufac), Sales (Sales), Services (Serv), Public administration and education (PubA.Edu)

Figure 4 displays the mean location of each class cohort according to the first two factorial coordinates of the MCAs. Since the MCAs are country- and cohort-specific, only the distances among social classes in the same cohort can be interpreted (dotted lines). These planes are the best possible two-dimensional representation of Latin American social spaces as operationalised here. The sizes of the points are proportional to the within-cohort percentage of couples (as in Table 4). The background lines are separated by one standard deviation to indicate the statistical significance and substantial importance of the differential positions of the classes.

In the figure, the five *probable social classes* form a J-shaped curve. The upper-left part of the social space is empty because Latin American societies do not have a social class with low economic capital and high cultural capital. I have added the CFR for the first (underlined number) and the last cohort to jointly show the processes of class consolidation and fertility decline. Significant and sustained differences across the CFR of distal classes reflect the divergent contexts in which couples make decisions regarding fertility. The similarity in the CFR of socially close classes reflects the reverse.

From left to right, the first class that appears is the lowest (red). This group is composed of couples with no education who were engaged in primary activities as self-employed workers in rural areas. This class also had the lowest levels of access to basic services (electricity, TV, piped water), although their homeownership rate was not the lowest across classes. Among the last cohort, the CFR for this group was above 4.7 in all six countries, except in Chile. In general, heterogeneity in the CFR of this class across countries increased over time. The CFR of women in the lowest class was between 6.9 in Bolivia and 8.3 in Colombia for the first cohort, but was between 3.5 in Chile and 7.2 in Bolivia for the last cohort.

The right side of the social space shows urban couples. This population is divided into four classes: low (orange), lower-middle (green), upper-middle (blue), and upper class (purple). The positioning of the low class below the lowest class on the vertical axis does not mean that the couples in the latter group had lower levels of cultural capital. This apparent difference is just a consequence of presenting the social space in a two-dimensional way.

The couples in the low class (orange) had either no education or primary education only. They tended to work in construction and manufacturing. In terms of their place of residence, the couples in this class were predominantly living in urban areas; but in countries like Bolivia, Colombia, and Mexico, some couples in the low class were living in rural areas. Their CFR was above six in all countries for the first cohort, and it decreased to between 3.1 in Chile to 6.0 in Bolivia for the last cohort. Given the relative size of the lowest and low classes combined, most of the change in aggregate fertility came from the changes in these two groups.

Most of the couples in the lower-middle class had primary education and were working in unskilled service, construction, or manufacturing jobs. The couples in this class had the lowest rates of homeownership. Their fertility started at levels between 4.6 in Brazil and 6.7 in Colombia in the first cohort and then declined to levels below 4.0 in all countries except Bolivia. Along with their counterparts in the low class, the couples in the lower-middle class were likely to be domestic migrants who moved from rural areas to intermediate/small cities searching for better working conditions in response to the weakening of agricultural production (Jelin 1977; Palloni et al. 1996). Census data do not allow me to track these moves, but the literature describing the urbanisation process in Latin America suggests that people who migrated from rural to urban areas joined these classes (Delgado-Wise 2014; Ducoff et al. 1965; Portes 1989; Rodríguez Vignoli 2004). Migrants to Santiago de Chile may be a slight exception to this pattern, as migrants in the city of Santiago had better socio-economic conditions than non-migrants (Balan 1969).

Most of the couples in the upper-middle class had secondary education, and some had university degrees. They were associated with non-manual occupations and were mostly living in urban areas. Compared to the previous three groups, the couples in this class had significantly higher levels of access to basic services and cultural resources. Access to water and electricity was nearly universal among the couples in this class, and a large proportion of them had completed secondary education. Their CFR was between that of the couples in the lower-middle and the upper classes. In Brazil, Colombia, and Chile, the CFR of this group decreased substantially across cohorts, converging to levels of around 2.5 children per woman. For the other three countries, the lowest observed CFR ranged from 3.1 in Paraguay to 3.8 Bolivia.

Most of the couples in the upper class had high educational levels, were living in a capital city, and had non-manual jobs in the service, education, or public administration sectors. As they were primarily wage/salary employees or employers, they had relatively high levels of social status, economic resources, and cultural capital. Unlike the lower classes, this group was relatively homogeneous in terms of size and CFR across the six countries, especially among the last cohort. The CFR for the couples in this class ranged from 2.1 in Brazil to 2.9 in Bolivia and Paraguay. Thus, the upper class could be characterised as a small, low-fertility class that displayed substantial levels of convergence across the six countries over time.

The relative sizes of and the persistent distances among the *probable social classes* reflect the dynamics of class consolidation. Smaller classes are more tightly enclosed than larger classes. Given the combination of the varying socio-economic conditions in each class and the social distances among them, moving between distal classes across generations is more difficult (less likely) than moving across classes that are in closer proximity. It is unlikely that the small increases observed in the upper classes across cohorts were driven by the incorporation of men and women who were born to couples in the lowest or low classes. These two groups were separated from the upper classes by more than two standard deviations in both directions, which means that their children were born in very distinct geographical areas and grew up in substantially disadvantaged opportunity structures in terms of access to economic and cultural resources. Couples in the lowest and low classes were separated from upper-class couples by, on average, at least 3.4 and 2.5 standard deviations, respectively. (Refer to Table 8 for the full list of between- and within-class mean distances).

To summarise, the CFR was closely associated with locations in the J-shaped social space. Despite the decline in fertility, this relationship lasted over time. For the couples of the first birth cohort, large families (seven to eight children) are displayed on the left side of the social space, whereas medium and small families (three to six) are shown on the right. The distribution of children ever born among couples on the right-hand side tracks the volume of cultural capital (bottom to top, large to small families). Differences along this dimension are smaller than those of the first dimension. A similar association can be seen for couples in the last cohort, i.e. large families are displayed on the left-hand side of the social spaces and small families are shown on the right side. However, differences along the second dimension are larger among couples of the last cohort than among couples of the first cohort given the increased relevance of cultural resources in differentiating social classes and shaping fertility outcomes. On the right side of the plots, the CFR ranges from six among the low classes to two among the upper classes.

Table 5 illustrates the class differences in the timing of childbearing by class and cohort. Despite some exceptions, the variation patterns are similar across the six nations. As a robustness check, Table 9 displays the distribution of women by parity levels, including women without children. Class variations in parity distribution are consistent with the following interpretations.

**Table 5:** Mean age at first and last birth by *probable social class* for the first and last cohort

Country	Probable social class									
	Lowest		Low		Lower middle		Upper middle		Upper	
Birth cohort	C1	C4	C1	C4	C1	C4	C1	C4	C1	C4
*Bolivia*										
First birth^1^	27.6	27.5	27.2	25.3	26.3	24.5	25.0	25.8	26.5	26.7
First birth^2^	24.1	22.9	23.6	22.6	24.1	22.4	23.9	23.6	25.1	25.0
First birth^3^	24.1	23.4	23.9	22.4	23.8	22.0	23.2	23.3	24.7	24.8
Last birth	37.6	37.8	37.3	35.6	36.0	34.1	34.6	33.3	33.6	33.2
*Brazil*										
First birth^1^	25.4	25.7	25.6	25.0	25.4	25.5	25.1	25.4	26.5	27.3
First birth^2^	22.8	21.4	22.9	20.6	23.2	22.1	23.2	22.4	25.1	25.8
First birth^3^	22.4	21.2	22.5	20.5	22.9	22.1	23.0	22.4	25.0	25.8
Last birth	37.1	34.5	36.1	31.7	33.7	30.9	32.7	30.5	32.8	31.4
*Chile*										
First birth^1^	26.4	25.7	26.5	25.1	26.1	25.7	25.8	26.3	26.5	27.5
First birth^2^	24.5	20.6	24.5	21.4	24.0	22.5	24.3	23.5	24.3	25.2
First birth^3^	24.1	20.5	24.0	21.4	23.6	22.4	23.8	23.5	24.1	25.2
Last birth	37.7	31.7	36.3	31.8	33.8	32.0	34.0	32.2	32.4	33.1
*Colombia*										
First birth^1^	25.6	25.5	25.8	25.1	24.9	25.3	24.8	26.5	25.4	28.8
First birth^2^	23.1	21.5	23.8	21.8	23.8	21.9	23.4	24.2	24.3	26.6
First birth^3^	22.4	21.3	23.2	21.6	23.1	21.8	22.8	24.2	23.9	26.5
Last birth	37.3	33.3	36.4	31.7	36.0	31.7	35.3	32.0	33.5	33.1
*Mexico*										
First birth^1^	25.4	25.3	25.3	24.6	25.0	24.2	25.0	24.2	25.8	26.3
First birth^2^	22.6	20.7	23.1	20.4	23.7	21.1	24.0	21.1	24.8	24.3
First birth^3^	22.1	20.4	22.5	20.2	23.0	20.9	23.4	21.0	24.3	24.2
Last birth	37.4	34.8	37.1	33.4	35.8	31.4	35.4	31.2	34.6	31.8
*Paraguay*										
First birth^1^	25.9	25.4	26.1	25.1	26.1	25.0	25.7	26.2	26.8	27.2
First birth^2^	23.7	22.8	24.4	22.8	24.6	23.4	25.0	25.0	26.3	26.2
First birth^3^	23.1	22.4	23.9	22.5	24.0	23.1	24.5	24.9	26.0	26.1
Last birth	38.6	36.7	36.1	34.8	35.5	33.6	33.6	33.4	34.2	33.5

*Note*: ^1^age at first birth based on the age of the oldest child living in the household (strongly upward bias), ^2^ age at first birth among women with complete birth histories (upward bias due to selection), ^3^ imputed age at first birth using matching process (reduced bias, still present)

The MAFB displays three interrelated patterns: (1) a positive association with class, (2) increasing between-class heterogeneity across cohorts, and (3) divergence over time among *probable social classes*. Women in the upper classes tended to have their first child later than women in the other classes. Because the MAFB declined for couples in the lower classes and increased or remained high for couples in the upper classes, cross-class differences in the age at first birth were more significant in the last cohort than in the first cohort. The MALB correlated negatively with social class. Across cohorts, this correlation weakened due to sharp declines in the MALB among couples in the lower classes.

These patterns of changes in fertility timing suggest that social changes had a differential association with the fertility outcomes of each social class. This result is consistent with the idea of fertility as a reproduction strategy closely linked to the sources and conversion rates of capitals (Bourdieu 1994; Torrado 1981). Education became more valuable over time as the national economies required better-trained workers living in large cities. The potential effects of such changes on the timing of childbearing were more likely to operate among the couples in the upper classes. These couples were the ones who had the greatest need to convert their economic capital into cultural capital through formal education, i.e. to spend more time in the educational system before having children. Meanwhile, the diversification of the labour market in the cities opened up new employment opportunities for men and women from the lower-middle class (some of whom migrated from rural areas), but these new positions did not require tertiary education. Lower-middle-class women were joining the labour force both formally and informally, potentially in response to their family’s financial needs. This shift may have contributed to the decline in fertility (Adserà and Menendez 2011; Schkolnik and Chackiel 2004). On the other side of the social spectrum, among couples in the lower classes, sources of capital continued to be scarce (due to the lack of basic services and limited access to education) and less valuable (due to economic shifts favouring the tertiary sector), which led them to have fewer children and to stop childbearing earlier.

These divergent trends in complete fertility and the timing of childbearing also suggest that access to modern contraception and FPPs varied across social classes. The women in the lowest and low classes experienced substantial declines in the CFR, an acceleration in the transition to parenthood, and a sharp decline in the age at last birth. These changes may be attributable to a lack of access to FPPs, hence recourse to, or forced sterilisation, as is common in rural areas (Bronfman et al. 1986; Caetano and Potter 2004). By contrast, couples in the upper classes delayed the transition to parenthood and shortened their childbearing period by maintaining a stable age at last birth. This sustained delay in the transition to childbearing is likely attributable to the use of contraceptive methods other than sterilisation, as has been observed among socially privileged groups across different historical contexts (Knodel and Van de Walle 1979).

Predictions from the quality–quantity trade-off hypothesis—which states that fertility declines because women prefer to have fewer children so they can invest more resources in them—are apparent for upper-class couples. Upper-class couples have both the dispositions and the resources to opt for a family size that maximises their investment in childrearing. Such predictions seem untenable for the rest of the social spectrum. Couples in the lower classes had minimal access to economic and cultural resources; it thus is doubtful that they were, in the first instance, seeking to maximise the transmission of intergenerational resources. The rationale for fertility decline among the lower classes was more likely the financial constraints that macroeconomic changes imposed on their lives than it was a means–ends calculation centred on maximising their offspring’s well-being.

Similarly, the opportunity cost hypothesis—which argues that women choose joining the labour force over childbearing—is plausible among upper-class women only. It is more likely that both a higher opportunity cost for entering the labour market and the disposition to identify such a cost (rational calculation) and to delay the first birth in response were present in educated women living in urban areas, but not in uneducated women (low and lower-middle classes) living in the countryside (lowest class). According to the data presented here, only upper-class women delayed the first birth, whereas women from lower classes accelerated this transition over time. Van de Kaa (1996) points to this mismatch when discussing demand-oriented explanations of fertility decline:

Important findings appear to be that there is a strong interaction between [the] quantity and quality of children although they are not close substitutes […]. The central problem of the narrative, however, is that it cannot be anchored firmly in what we know about the way things happen in this world. While one might, with some imagination, place the story in the context of middle-class America, it is difficult to see how it could apply in a less developed country where time is abundant and ‘consumer choice’ largely absent. (van de Kaa 1996, p. 410)

Finally, the similarities in the fertility outcomes among the socially close classes and the differences in the fertility outcomes among the socially distal classes suggest that any diffusion of social norms regarding the timing of family formation and family size is more likely to occur within than between classes, or, at most, between socially close classes. The social distance between the lower and the upper classes (on average, at least 1.7 standard deviations) and the fact that they tend to live in geographically different places (rural areas, urban areas, and large cities) imply that social interaction and social contagion/imitation across classes are very unlikely.

## Conclusions and Discussion

As a result of dramatic socio-economic changes in Latin America, cohorts born between 1920 and 1965 faced very different family formation contexts. An under-developed region was transformed into a developing region through the implementation of far-reaching and long-lasting social and economic policies that exacerbated social inequalities. Fertility decline was a component of this transformation and followed class-specific trajectories that contributed to the consolidation of highly unequal social stratification systems. I used a multidimensional and relational definition of social class to describe these processes and to reinterpret some of the classical theories of fertility change. With this approach to social class—along with a theoretical assumption regarding class-specific dispositions—I have been able to identify three main aspects of the relationship between fertility and class: (a) the enduring connection between social stratification and fertility, (b) the coexistence of diverse fertility decline trajectories, and (c) the dual role of social distances in promoting and preventing ideational change.

The complete fertility levels across all of the cohorts in the six Latin American countries studied here were closely tied to the overall distribution of social classes within the social space. Over time, unequal socio-economic development across countries led to an unequal—yet generalised—fertility decline, which was accompanied by changes in the countries’ class compositions. Class consolidation consisted primarily of the growth of the low and lower-middle classes, the two groups with the most significant changes in the CFR and the MALB. These two groups were, consequently, the main contributors to fertility decline.

The results show that the lower- and upper-class couples experienced divergent fertility transitions. While the upper-class women delayed the first birth, kept the childbearing period relatively stable, and experienced the smallest CFR declines, the lower-class women accelerated the first birth, shortened the childbearing period, and reduced their complete fertility. Middle-class couples deserve their label not only because of their socio-economic attributes, but because the characteristics of their fertility transition were between those of the lower- and upper-class couples. The lower a couple’s class, the more likely the female partner was to follow a stopping strategy to reduce the number of children, potentially through sterilisation. Class differences in access to (voluntary or forced) sterilisation may partly explain this trend, because this practice is highly effective for stopping childbearing, and its use was more prevalent among women of lower than of higher socio-economic status. At the other end of the class spectrum, the higher a couple’s class, the more likely the female partner was to delay the first birth and achieve a relatively small family size by age 40. This fertility trajectory tends to be associated with contraceptive methods other than early sterilisation.

The class-specific trajectories observed here call into question the predictions of classical theories of fertility decline that have focused on interpreting independent (pure) associations (effects) of socio-economic variables on fertility. Because social classes are multidimensionally constructed, socio-economic changes have class-specific associations with fertility and fertility timing. These class-specific relationships are only evident if the focus is on the intersection of individuals’ material conditions of existence (their class) and the class dispositions they generate. To further illustrate this point, let us consider class differences in educational attainment, female labour force participation, and access to modern contraception from the dual perspective of the social class positions and social class dispositions.

Educational attainment did not grow substantially for the lower classes, yet these three groups experienced significant declines in the number of children ever born. Given the sizes of these social classes, their contributions to the overall fertility decline were the largest, which suggests that education did not play a central role in the overall changes in complete fertility at the country level. The contrary pattern was observed for upper-class couples: the proportions of these couples with secondary and tertiary education increased substantially across cohorts, which undoubtedly contributed to their lower fertility and delayed transitions to parenthood. Their contribution to the overall fertility decline was small, given their reduced size as a class. It is important to recall that there was a spatial factor in these classes, as the couples in the lower classes were mostly living in rural areas, whereas the upper-middle- and upper-class couples were mostly living in urban areas and capital cities, respectively. These spatial differences explain not only the differences in the couples’ opportunity to access formal education and non-manual (clerical) jobs, but the differential returns they had from gaining educational credentials vs. entering parenthood. This is not to negate the role of education on fertility change, but to underline the long-standing idea that the role of education on societal change is context-dependent, or for the same matter, class-dependent (National Research Council 1999, Chapter 4)

The finding that women in the lower and middle classes had similar labour force participation rates but very different fertility levels suggests that the association between these two variables was also class-specific. The conflict between childrearing and labour force participation was likely to be more acute in urban areas, especially among recently arrived migrant couples from rural areas. In cities, daily commutes to work are necessary, whereas in rural areas, the household and the place of work are more likely to coincide (Hervitz 1985; Schockaert 2005). Moreover, multigenerational households are more prevalent in rural than in urban areas, which suggests that kinship support for childbearing and childrearing may favour the coexistence of relatively high fertility and labour force participation (Bongaarts 2001; De Vos 1995).

Modern contraceptive methods were not available to the women of the first two birth cohorts. However, even in these cohorts, upper-class women had lower completed fertility and later transitions to motherhood than women in other classes. A persistent disposition towards having a smaller family, along with the material means to practice birth control effectively (without modern contraception), may explain the similarities in the fertility outcomes of the upper-class couples across the six countries and over time. At the other end of the class spectrum, even among the last cohort in countries with strong family planning programmes, lower-class couples did not display fertility outcomes that are consistent with the use of contraceptive methods such as condoms, the pill, or the intrauterine contraceptive device.

There are two potential explanations for this difference between the upper and lower classes. First, the lower classes were less likely to have access to modern contraceptive methods because they were living in remote rural areas with less access to basic services; consequently, it is unlikely that the fertility decline observed among them is attributable to reproductive health services. See the work of Caetano and Potter (2004) for Northeast Brazil, Svallfors and Billingsley (2019) for Colombia, and Brofman, López, and Tuirán (Bronfman et al. 1986) for Mexico, and Vidal-Zeballos (1994) for Bolivia. Second, the disposition of lower-class couples to incorporate the use of modern contraception into their reproductive lives may differ from that of the upper classes. Studies on teenage childbearing in Colombia have shown that low-SES teenagers report negative attitudes towards the use of condoms, as it can raise issues of trust with their partners (Flórez and Soto 2007). Studies conducted elsewhere have also shown that modern contraception can be used not only to limit fertility, but also to control birth spacing in contexts with rigid social norms on these issues (Bledsoe et al. 1994).

The role of ideational change and social contagion is also subject to class-specific conditions. La Ferrara et al. (2012) used the 1991 Brazilian census and spatial information on the expansion of the largest Brazilian television network during the early 1980s to explore the role of ideational change on fertility decline. The authors reported an overall negative causal effect of television on fertility. They attributed this effect to the fact that Brazilian soap operas display small families (two children) as an ideal model for acquiring social mobility. However, according to the evidence presented here, it is more likely that this effect operated on middle- and upper-class couples than on couples in the lowest classes given the lack of access of the latter to electricity service and television. Indeed, it should be noted that among the couples in the upper classes, the most common parity level was two children after the second cohort, a cohort who did not benefit from the expansion of television. Ideational change towards a preference for smaller families is more likely to operate among people with both (1) access to sources promoting these types of families (television), and (2) the socio-economic motivation to incorporate such changes.

Ideational change towards a preference for smaller families can also spread across social groups through social interaction. Studies on fertility transitions in Europe and in Latin America have argued that, over time, fertility outcomes converge across classes as upper-class behaviour spreads to the lower classes (Dribe et al. 2017; Schmertmann et al. 2005). The evidence presented here contradicts this hypothesis. The durability of the distance across classes in the social space, the degree of residential segregation by class (rural vs. urban, but also within cities between centric and peripheral neighbourhoods: *villas miseria*, *barrios*, *barrios de invasión*, *favelas*), and the similarities of fertility outcomes within classes that we observed suggest that social contagion is more likely to occur within than between classes. In Latin America, the concentration of resources in large cities and the residential segregation that characterises urban development make within-class interactions more likely to occur, and increase the chances that these interactions will be more instrumental in transmitting ideas than between-class interactions. Moreover, the historical isolation of rural areas, especially those affected by violence, reduces the chances of between-class interactions taking place (Castro Torres and Urdinola 2019).

Consequently, it is unlikely that the fertility decline among lower-class couples was driven by their intentions to replicate the fertility behaviour of upper-class women. Indeed, these patterns of decline do not look similar. Analogously, the strong similarities observed in the fertility outcomes of the middle classes (lower middle and upper middle) implies that their physical proximity (urban areas), as well as their social proximity, may have played a role in making them demographically similar. The so-called leaders/forerunners of demographic change and their corresponding social imitators may exist, but primarily within each class, or, at most, between socially close classes.

All in all, these analyses suggest that classical explanations of fertility decline must be used in conjunction with a multidimensional and relational definition of social class. Otherwise, such explanations fall short in accounting for demographic changes, especially among couples in the lowest, low, and lower-middle classes, who were the most critical contributors to fertility decline and class consolidation.

By letting the social space be contingent on the historical context and by putting the relative position of collectives in the social space as an explanatory category, both micro- and macro-level factors are inductively incorporated in our understanding of societal change. Because it focuses on the historical experiences of cohorts and of *probable social classes*, rather than on the correlation between dependent and (rarely) independent variables, this approach forces the accounts to be about the actual makers of demographic and societal change (Emirbayer 1997; Lieberson and Horwich 2008). This distinction is not minor, as it implies the construction of scientific narratives in which the subjects of the statements are not variables; e.g. education does *X*, unemployment prevents *Y*, migration causes *Z*, but social groups defined in terms of birth cohorts, and classes (Ryder 1965).

The limitations of this work underline lingering questions. First, there is a dearth of qualitative work on fertility preferences and intentions—and the realisation thereof—in Latin America. This type of work can shed light on how class conditions relate to fertility outcomes and assess the extent to which quantitative approaches capture these relations correctly. Second, more detailed datasets such as those of the Demographic and Health Surveys or country-specific fertility surveys can be used to examine the influence of domestic and international migration on fertility. These two demographic dynamics were pervasive after 1950–1960, and both are very likely to affect family dynamics (Landale and Oropesa 2007).

## Figures and Tables

**Fig. 6:**
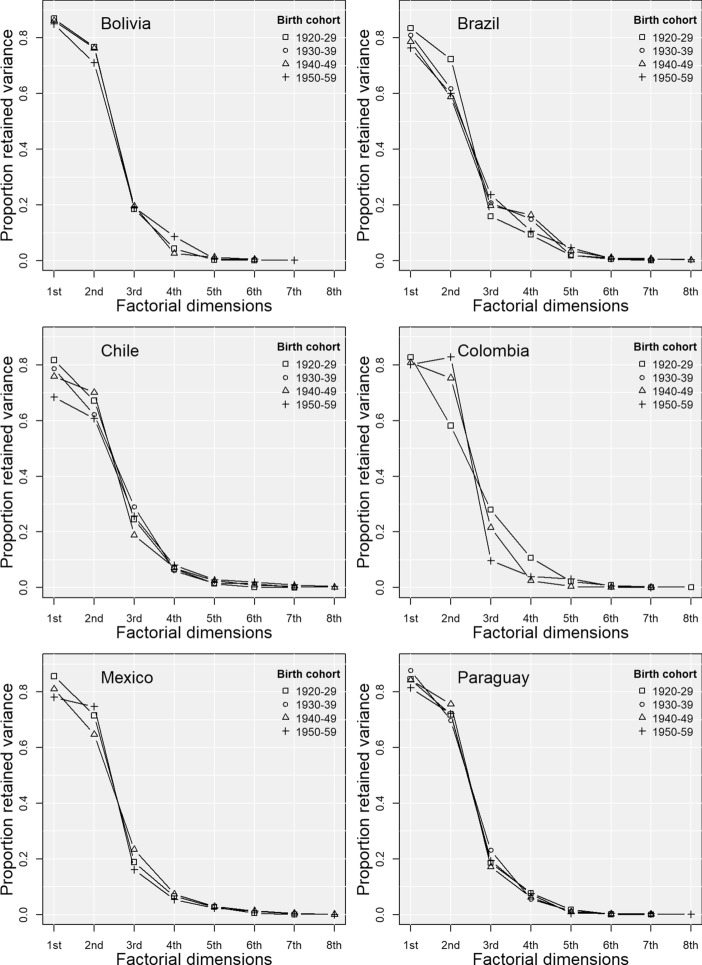
Proportion of remaining explained variance. *Note*: The number of factorial dimensions equals the number of categories (Table III) minus the number of variables (9). Proportions are calculated according to Greenacre and Blasius’ (2006) formula

**Table 8:**
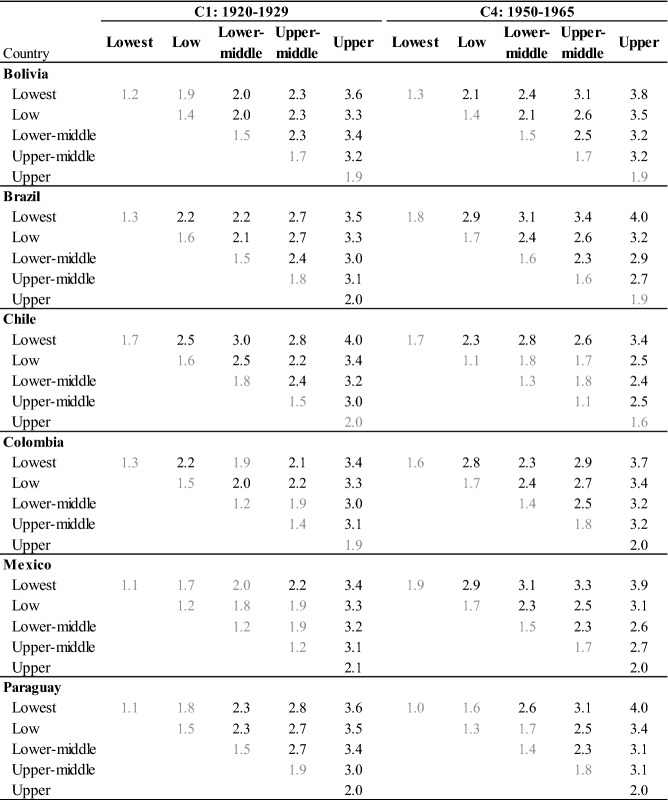
Standardised mean social distance among probable social classes

*Note*: Social distance is calculated using the first four factorial coordinates of the multiple correspondence analyses. Mean values are divided by the standard deviation. Values below 1.96 are written in grey, while values above 1.96 are written in black

## Appendix

Figure 5 displays the heterogeneity in fertility and developmental trajectories across selected countries in Latin America from 1950 to 2010. Labelled countries are included in the analysis, and the grey lines correspond to other Latin American nations that, due to data limitations, could not be included in the analysis (Figs. 6, 7, 8, 9, 10, 11, 12, 13).

Table 6 addresses potential concerns about the sample selection. This table shows that: (1) excluding separated, divorced, and never-married women can bias the results; but also the conclusions are unlikely to change if I included them because differences in the fertility outcomes are small, except for never-married women, whose contribution to the total births is minimal (slightly less than 4%); (2) excluding women due to the absence of a partner is unlikely to produce systematic bias.

**Table 6:** Fertility indicators of women in the analytical sample and women not included in the analysis by reason of exclusion

		Not included due to:					
Variable	Analytical sample	Missing information		Marital status			Overall
		Woman	Partner	Separated or divorced	Widowed	Never married	
*Panel A: fertility indicators*							
Mean children ever born	5.3	5.7	4.9	3.7	5.0	1.8	4.7
Mean age at (years)							
First birth	25.7	25.8	25.7	25.3	24.8	27.4	25.8
Last birth	34.6	35.1	33.8	31.0	31.9	32.5	33.8
Children ever born (%)							
Zero	3.1	3.7	4.2	3.9	3.6	43.8	8.2
One	5.9	5.3	7.1	13.8	8.1	18.8	8.3
Two	13.9	10.3	14.3	21.4	12.7	11.0	14.0
Three	15.9	12.9	15.7	19.3	14.4	7.6	15.0
Four	12.4	11.3	12.7	13.0	12.2	5.2	11.6
5–6	17.6	19.3	18.0	14.6	19.8	6.9	16.4
7+	31.1	37.2	28.0	13.9	29.3	6.7	26.5
Total	100.0	100.0	100.0	100.0	100.0	100.0	100.0
*Panel B: women’ socio-economic characteristics*							
Educational attainment (%)							
No education	60.4	68.9	56.4	43.7	67.9	51.7	58.0
Primary	26.5	22.4	29.7	32.5	23.7	28.6	27.5
Secondary	10.9	7.2	11.4	18.8	7.3	15.6	11.8
Tertiary	2.3	1.4	2.5	5.1	1.1	4.1	2.7
Total	100.0	100.0	100.0	100.0	100.0	100.0	100.0
*Place of residence (%)*							
Capital city	25.4	19.0	24.9	36.2	25.8	30.3	26.5
Rural	35.0	43.2	27.6	11.4	29.5	24.8	30.6
Urban	39.6	37.8	47.6	52.4	44.8	44.9	42.9
Total	100.0	100.0	100.0	100.0	100.0	100.0	100.0
							0.0
*Panel C: total numbers and contribution to total births*							
Number of women	17,68,500	49,157	5,86,050	2,77,691	1,80,830	3,03,150	31,65,378
% contribution to births	62.7	1.9	19.6	6.9	6.2	3.7	100.0
% among total women	54.8	1.7	19.4	6.7	5.8	11.6	100.0
% among ever married/in union	62.0	1.9	22.0	7.6	6.5		100.0

*Note*: The mean ages at first and last birth are computed as the difference between the age of the woman and the age of the oldest and the youngest own child in the household. The bias of these figures is discussed and partially corrected in the paper for women in the analytical sample

Women who were in a couple, but for whom no information about their partner was available, make up 19% of the pooled sample. These women did not differ substantially from the women in the analytical sample in terms of their fertility indicators. Their fertility was slightly lower (4.9 vs. 5.3 children ever born), their mean age at first birth was the same (25.7), and their mean age at last birth was slightly lower (33.8 vs. 34.6). The socio-economic characteristics of these women also do not suggest a systematic bias. This group was not overrepresented in either the highest or lowest categories of educational attainment, or in the capital cities or rural areas.

Table 7 presents a summary of bivariate linear probability models by country that predict the proportion of women with incomplete birth histories based on their socio-economic characteristics. This table shows strong, negative correlations between socio-economic status and the prevalence of incomplete birth histories. These negative correlations mean that the upward bias in the age at first birth is likely to be more pronounced among low-SES than high-SES women (Tables 8, 9).

**Table 7:** Bivariate associations between socio-economic variables and the proportion of incomplete birth histories by country

Variable	Country											
	Bolivia		Brazil		Chile		Colombia		Paraguay		Mexico	
	Coeff.	*p* value	Coeff.	*p* value	Coeff.	*p* value	Coeff.	*p* value	Coeff.	*p* value	Coeff.	*p* value
*Woman’s educational attainment*												
Constant	0.81	0.000	0.64	0.000	0.72	0.000	0.75	0.000	0.76	0.000	0.77	0.000
Primary (ref: No education)	− 0.14	0.000	− 0.28	0.000	− 0.20	0.000	− 0.21	0.000	− 0.24	0.000	− 0.25	0.000
Secondary (ref: No education)	− 0.37	0.000	− 0.40	0.000	− 0.39	0.000	− 0.44	0.000	− 0.46	0.000	− 0.47	0.000
University (ref: No education)	− 0.53	0.000	− 0.50	0.000	− 0.51	0.000	− 0.56	0.000	− 0.56	0.000	− 0.59	0.000
*Men’s educational attainment*												
Constant	0.82	0.000	0.64	0.000	0.73	0.000	0.74	0.000	0.77	0.000	0.77	0.000
Primary (ref: No education)	− 0.10	0.000	− 0.27	0.000	− 0.20	0.000	− 0.20	0.000	− 0.21	0.000	− 0.24	0.000
Secondary (ref: No education)	− 0.29	0.000	− 0.38	0.000	− 0.35	0.000	− 0.40	0.000	− 0.40	0.000	− 0.45	0.000
University (ref: No education)	− 0.48	0.000	− 0.46	0.000	− 0.45	0.000	− 0.45	0.000	− 0.51	0.000	− 0.53	0.000
*Place of residence*												
Constant	0.59	0.000	0.42	0.000	0.47	0.000	0.45	0.000	0.50	0.000	0.40	0.000
Rural areas (ref: Capital city)	0.24	0.000	0.27	0.000	0.29	0.000	0.32	0.000	0.29	0.000	0.38	0.000
Urban areas (ref: Capital city)	0.09	0.000	0.14	0.000	0.10	0.000	0.13	0.000	0.09	0.000	0.16	0.000
*Ownership of the dwelling*												
Constant	0.75	0.000	0.55	0.000	0.54	0.000	0.62	0.000	0.63	0.000	0.66	0.000
Renting (ref: Owned)	− 0.09	0.000	− 0.03	0.000	− 0.03	0.000	− 0.03	0.000	0.00	0.482	− 0.14	0.000
Provided (ref: Owned)	− 0.01	0.582	0.08	0.000	0.13	0.000	0.13	0.000	n.a	n.a	n.a	n.a
*Water supply*												
Constant	0.58	0.000	0.46	0.000	0.49	0.000	0.56	0.000	0.54	0.000	0.41	0.000
Piped shared (ref: Piped exclusive)	0.17	0.000	0.24	0.000	0.19	0.000	0.14	0.000	0.19	0.000	0.15	0.000
No piped water (ref: Piped exclusive)	0.24	0.000	0.26	0.000	0.30	0.000	0.22	0.000	0.24	0.000	0.34	0.000
*Woman’s position at work*												
Constant	0.72	0.000	0.51	0.000	0.47	0.000	0.51	0.000	0.60	0.000	0.53	0.000
Employee(r) (ref: Self-account)	− 0.21	0.000	− 0.08	0.000	− 0.06	0.000	− 0.09	0.000	− 0.16	0.000	− 0.11	0.000
Inactive (ref: Self-account)	0.05	0.000	0.08	0.000	0.11	0.000	0.15	0.000	0.06	0.000	0.18	0.000
*Men’s position at work*												
Constant	0.58	0.000	0.44	0.000	0.44	0.000	0.64	0.000	0.54	0.000	0.48	0.000
Employer (ref: Self-account)	0.20	0.000	0.17	0.000	0.14	0.000	0.02	0.066	0.15	0.000	0.24	0.000
Employee(r) (ref: Self-account)	0.10	0.000	0.08	0.000	0.10	0.000	− 0.04	0.000	0.06	0.000	0.08	0.000
*Man’s economic sector*												
Constant	0.82	0.000	0.69	0.000	0.72	0.000	0.76	0.000	0.78	0.000	0.80	0.000
Manufacturing (ref: Agriculture)	− 0.13	0.000	− 0.22	0.000	− 0.20	0.000	− 0.23	0.000	− 0.19	0.000	− 0.23	0.000
Construction (ref: Agriculture)	− 0.09	0.000	− 0.09	0.000	− 0.12	0.000	− 0.13	0.000	− 0.10	0.000	− 0.18	0.000
Services (ref: Agriculture)	− 0.21	0.000	− 0.25	0.000	− 0.26	0.000	− 0.25	0.000	− 0.25	0.000	− 0.35	0.000
Sales (ref: Agriculture)	− 0.18	0.000	− 0.22	0.000	− 0.23	0.000	− 0.22	0.000	− 0.22	0.000	− 0.30	0.000
Clerical and education (ref: Agriculture)	− 0.25	0.000	− 0.25	0.000	− 0.27	0.000	− 0.28	0.000	− 0.30	0.000	− 0.37	0.000

*Note* Incomplete birth histories are defined as birth histories in which the total number of children ever born is larger than the total number of own children recorded by the census. Regression models were run separately by country and variable

**Table 9:** Women’s distribution by parity level according to *probable social class* for the first and last cohort

Country	Lowest		Low		Lower middle		Upper middle		Upper	
Birth cohort	C1	C4	C1	C4	C1	C4	C1	C4	C1	C4
*Bolivia*										
Zero	3	2	3	1	3	1	3	2	4	3
One or two	8	6	6	10	11	14	12	25	18	38
Three or four	13	12	11	22	18	36	25	48	42	50
Five or more	76	79	80	67	68	49	60	25	35	9
Total	100	100	100	100	100	100	100	100	100	100
*Brazil*										
Zero	5	3	5	3	5	4	5	4	7	7
One or two	9	14	13	25	26	43	30	48	37	60
Three or four	13	25	17	39	28	40	32	40	34	31
Five or more	73	58	65	33	41	13	33	7	21	1
Total	100	100	100	100	100	100	100	100	100	100
*Chile*										
Zero	2	2	2	2	4	3	3	3	4	4
One or two	8	29	14	35	25	43	21	49	31	48
Three or four	12	46	19	50	30	46	32	43	42	43
Five or more	78	24	64	13	42	8	45	5	23	5
Total	100	100	100	100	100	100	100	100	100	100
*Colombia*										
Zero	2	4	3	4	3	4	3	5	3	7
One or two	6	15	13	29	10	31	11	52	17	63
Three or four	10	34	17	44	16	47	19	38	33	28
Five or more	82	47	68	23	71	19	67	5	47	2
Total	100	100	100	100	100	100	100	100	100	100
*Mexico*										
Zero	7	3	6	2	7	2	6	2	7	3
One or two	6	8	8	9	12	20	14	22	17	44
Three or four	10	20	11	26	16	48	21	50	29	46
Five or more	77	69	75	64	65	29	59	26	48	6
Total	100	100	100	100	100	100	100	100	100	100
*Paraguay*										
Zero	3	1	4	2	3	2	3	2	3	3
One or two	8	8	15	13	19	24	29	35	24	38
Three or four	8	13	21	28	25	40	36	48	46	49
Five or more	81	78	60	57	52	34	32	15	27	10
Total	100	100	100	100	100	100	100	100	100	100

## Footnotes

1 Lack of comparability and missing information in the place of residence, occupation, and household assets prevent me from including Argentina, Ecuador, Peru, Uruguay, and Venezuela. Data are publicly available upon registration at: https://international.ipums.org/international/.

2 Refer to Table 6 for additional checks and discussions on the representativeness of the analytical sample.

3 As a robustness check, I computed the bivariate relationships between socio-economic characteristics and the proportion of incomplete birth histories. According to Table 7, the higher the socio-economic level and social status of a couple, the lower the proportion of incomplete birth histories. These results confirm that class differences in the mean age at first birth are likely to be underestimated.

4 An alternative presentation is these results is displayed in Fig. 6. This figure plots the proportion of the remaining variance that is explained by each factorial dimension.
